# *Actinobacillus hominis* osteomyelitis: First reported case in the English language medical literature

**DOI:** 10.1099/jmmcr.0.005030

**Published:** 2016-06-10

**Authors:** Gavin O’Neill, Andrew Ker, Aslam Mohammed, Anne Marie Karcher

**Affiliations:** ^1^​ Department of Orthopaedics, Southern General Hospital, Glasgow, UK; ^2^​ Department of Microbiology, Aberdeen Royal Infirmary, Aberdeen, UK

**Keywords:** *Actinobacillus*, *Actinobacillus hominis*, ciprofloxacin, laboratory identification, osteomyelitis, pasteurellaceae

## Abstract

**Introduction:**

*Actinobacillus hominis* is currently a rarely reported pathogen. It has previously been associated with respiratory tract infections and bacteraemia in debilitated patients. However, under-reporting may occur due to misidentification by commonly used laboratory bacterial identification systems. This case is, to the best of our knowledge, the first reported case of *A. hominis* osteomyelitis in the English language medical literature.

**Case presentation:**

A 37-year-old male presented with a painful foot. He had no previous foot problems, history of injury or animal contact. Osteomyelitis was confirmed by magnetic resonance imaging (MRI), and blood cultures were positive for Gram-variable bacilli. The organism was identified initially as *Pasteurella pneumotropica* by the local routine diagnostic laboratory and as a *Pasteurella* species by the UK National Reference Laboratory (Colindale, London, UK), using standard operating procedures at the time. It was finally identified as an *A. hominis* using 16S rRNA gene sequence analysis. Difficulties in the accurate identification of this organism remain current, as other biochemical identification systems have also resulted in misidentifications. The patient refused admission and intravenous antibiotics. He was successfully treated using an 8-week course of oral ciprofloxacin and amoxicillin based on antibiotic disc susceptibility testing resulting in clinical, serological and radiological resolution.

**Conclusion:**

Laboratories should maintain a high index of suspicion for *A. hominis* as several commonly used bacterial identification systems may not accurately identify the organism. Colonial morphology and absence of animal contact should prompt consideration of this organism in appropriate clinical situations. Oral ciprofloxacin and amoxicillin treatment was successful in this case.

## Introduction

The genus *Actinobacillus* belongs to the family *Pasteurellaceae*. Most species within the genus are animal pathogens with the exception of *Actinobacillus hominis* and *Actinobacillus ureae, *both of which are regarded as human pathogens. *A. hominis* is an oxidase-positive, Gram-negative bacillus, which grows as 1–2 mm greyish-white, non-haemolytic colonies on blood agar after overnight incubation in CO_2_. It is typically sensitive to ampicillin, chloramphenicol, ciprofloxacin, erythromycin, gentamicin, penicillin and tetracycline on antibiotic disc diffusion susceptibility testing. Infections with *A. hominis* have rarely been reported in the literature and have included respiratory tract infections and bacteraemias, mostly in patients with co-morbidities (Friis-Møller *et al*., 2001). However, under-reporting may be occurring due to difficulties with identification. Accurate identification is important to enable the recognition of emerging pathogens, the identification of risk factors, and differences in clinical and treatment outcomes for different organisms. Misidentification of organisms may also explain the absence of known risk factors in some apparent cases, for example in the absence of animal contact. Therefore, it is important that there is increased awareness of the potential for misidentification, with consideration given to further confirmatory testing. Here we report, to the best of our knowledge, the first case of *A. hominis* osteomyelitis in the English language medical literature.

## Case report

**Fig. 1.**
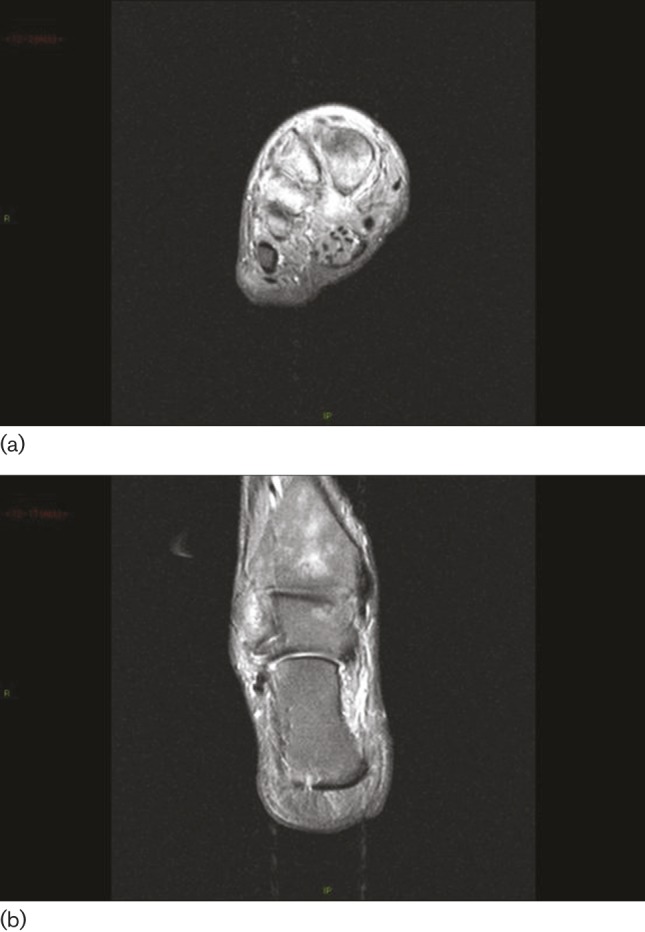
(a) Widespread bony oedema affecting the first, second, third and fourth metatarsals, with associated soft tissue oedema. (b) Widespread bony oedema also affecting several of the tarsal bones, the talus and the calcaneus, as well as the tibia.

**Fig. 2.**
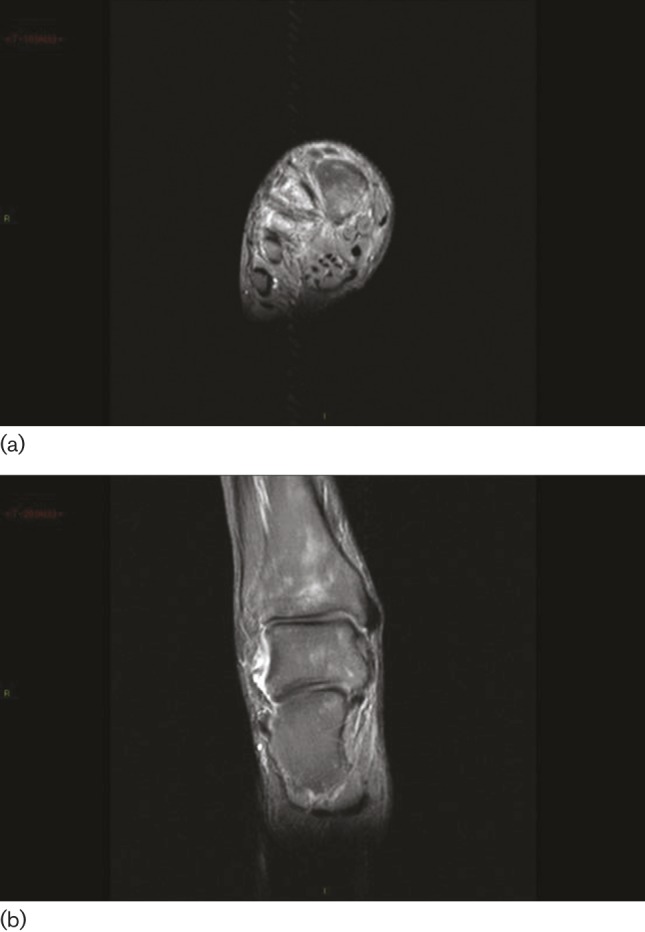
(a) Bone oedema within the fourth metatarsal and cuneiforms has subsided. (b) Non-specific patchy altered signal still present within the cuboid, calcaneus, talus and distal tibia, although this has improved.

A 37-year-old male presented to our Accident and Emergency department complaining of a swollen, painful foot. There was no history of previous foot problems, injury or animal contact. He had no respiratory symptoms. He had a past medical history of a head injury resulting in subsequent seizures. The patient was not diabetic or debilitated, and there was no other significant past medical or drug history. He left prior to completion of assessment, but re-attended 2 weeks later with increased pain, swelling and rubor of his foot, and had been vomiting for the previous 48 h. There were signs of significant systemic upset with pyrexia (temperature 38^o^C) and tachycardia (pulse 144 beats min^–1^), with a blood pressure of 119/83 mmHg. His white cell count was 13.6×10^9^ l^–1^ (reference range 4×10^9^–11.6×10^9^ l^–1^), predominantly neutrophils (11.1×10^9^ l^–1^), and his C- reactive protein was 267 mg l^–1^ (reference range <10 mg l^–1^). A clinical diagnosis of osteomyelitis was made, and this was confirmed radiologically by MRI scan (Fig. 1). T2-weighted images showed widespread osteomyelitis of the first to fourth metatarsals with extensive soft tissue oedema and oedema in the talus, calcaneum and distal tibia. Blood cultures collected at re-presentation showed Gram-variable bacilli after 48 h incubation, which grew on blood agar (Oxoid) as oxidase-positive, non-haemolytic, large greyish-white colonies after overnight incubation. The organism was identified by the local laboratory using API 20NE (bioMérieux), following the laboratory standard operating procedure at the time, as a *Pasteurella pneumotropica *(API 20NE profile 1220004). However, the colonial morphology and absence of a history of animal contact resulted in the organism being sent for further testing and confirmation to the UK National Reference Laboratory (Colindale, London). Gas chromatography identified the organism as a *Pasteurella* sp. However, further testing using 16S rRNA gene sequence analysis confirmed the identification as *A. hominis. *The patient repeatedly refused admission and was given an 8-week course of oral ciprofloxacin and amoxicillin, appropriate to the organism sensitivities. Within 48 h, the patient’s symptoms had settled. A follow-up MRI scan after 6 weeks of antibiotics demonstrated a marked improvement (Fig. 2), which correlated with clinical improvement in his symptoms and serial improvements in CRP. CRP at 10 weeks was 2 mg l^–1^ and his white cell count had returned to normal at 4.9×10^9^ l^–1^. He was discharged from further orthopaedic follow-up after his 6-month clinical review, as there were no clinical signs of infection. An X-ray of his foot taken 1 year after initial presentation, which followed an unrelated injury to his foot, showed no radiological evidence of infection. Therefore, the patient had clinical, serological and radiological evidence of successful treatment.

## Discussion

To the best of our knowledge, this is the first reported case of *A. hominis* osteomyelitis in the English language medical literature. The organism was confirmed by 16S rRNA gene sequence analysis of a blood culture isolate in the presence of clinical and radiological features of osteomyelitis.

As a principle, the most important consideration for the treatment of osteomyelitis is that the antibiotic concentration exceeds the MIC of the infecting organism at the site of the infection, regardless of the route of administration. However, to ensure systemic levels, treatment for osteomyelitis is often, at least initially, parenteral, and is usually prolonged (Rao *et al*., 2011). However, in this case, the patient refused hospital admission and intravenous antibiotics. Despite extensive bony changes seen on MRI of his foot and a significant degree of systemic upset at presentation with bacteraemia, this patient was successfully treated with an 8-week course of oral amoxicillin and ciprofloxacin to which the organism was shown to be sensitive on antibiotic susceptibility testing, resulting in clinical, serological and radiological resolution.

Accurate identification of organisms, especially from sterile sites, is important. The organism was initially misidentified as a *P. pneumotropica*, an organism that is endogenous to animals and has been associated with animal contact (Gadberry *et al*., 1984), and is a recognised cause of osteomyelitis (Frebourg *et al*., 2002; Gadberry *et al*., 1984; Sammarco & Leist, 1986). In contrast, *A. hominis* is said to be highly adapted to humans (Friis-Møller *et al*., 2001). Gas chromatography, which was part of the Reference Laboratory standard operating procedure at the time, is of particular value for organisms that are not easily identified by other methods. However, identification is dependent on the comparison of chromatograms for unknown organisms with those of known organisms. Misidentification of the organism has also been described using VITEK GNI+ and ID 32E (Friis-Møller *et al*., 2001) and the VITEK 2NH card (de Melo Oliviera *et al*., 2013) (both from bioMérieux). At the time of this report, *A. hominis* is not included in the databases for matrix-assisted laser desorption/ionization time-of-flight mass spectrometry performed using either a Microflex LT instrument (Bruker Daltonics) (Bizzini *et al., *2011) or VITEK MS (bioMérieux). *A. hominis* appears currently to present a diagnostic challenge. Therefore, increased awareness in routine diagnostic laboratories is appropriate so that, in relevant clinical circumstances, consideration may be given to further confirmatory testing using reference methods such as 16S rRNA gene sequencing.
